# Efficacy of the Modified Alvarado Scoring System and Radiologic Studies in Diagnosing Appendicitis in Children: A Retrospective Comparative Study

**DOI:** 10.7759/cureus.72873

**Published:** 2024-11-02

**Authors:** Ahmed Mnofala, Abdulhadi Tashkandi, Mohammad Halawani, Mohammed A Ahmed, Zuhair Hummaida, Alawi M Al-Mashhor, Ahmed Othman, Emad Uddin Siddiqui

**Affiliations:** 1 Emergency Department, Prince Mohammad Bin Abdulaziz Hospital, Madinah, SAU; 2 Emergency Medicine Department, Prince Mohammad Bin Abdulaziz Hospital, Madinah, SAU; 3 Pediatric Surgery Department, Prince Mohammad Bin Abdulaziz Hospital, Madina, SAU; 4 Emergency Department, Royal Albert Edward Infirmary, Wigan, GBR; 5 Pediatric Emergency Department, Prince Mohammad Bin Abdulaziz Hospital, Madinah, SAU

**Keywords:** appendicitis, diagnostic accuracy, modified alvarado score, tomography, ultrasound

## Abstract

Background: Appendicitis is a common diagnostic challenge in pediatric patients. Based on clinical and laboratory findings, the modified Alvarado scoring system (MAS) is commonly used to assess the likelihood of appendicitis. This study aims to evaluate the diagnostic performance of MAS compared to radiologic findings and its components in the diagnosis of appendicitis in children.

Methods: This retrospective study involved 177 pediatric patients with abdominal pain. The MAS, radiologic examinations (ultrasound), and physical examination findings were evaluated. Data on MAS, sensitivity, specificity, positive predictive value (PPV), and negative predictive value (NPV) were analyzed. Chi-square tests were used to determine significant differences between MAS and radiologic findings.

Results: The study included 92 males (52%) and 85 females (48%), with the majority (95, 53.7%) in the age group of 7-10 years. Of the 177 patients, 46 (26%) had positive radiologic findings for appendicitis. The MAS showed a sensitivity of 81%, a specificity of 69%, a PPV of 48%, and an NPV of 91%. A significant difference in the MAS was found between patients with positive and negative radiologic findings (p = 0.001). Compared with ultrasound, MAS showed a PPV of 39%, an NPV of 14%, a sensitivity of 48%, a specificity of 80%, an accuracy of 73%, and an odds ratio of 3.69. Rebound tenderness on physical examination also showed a significant association with appendicitis (p = 0.001).

Conclusions: This study highlights the potential of the MAS as a valuable tool for the initial assessment of appendicitis in children, demonstrating high sensitivity and NPV. Although it has a moderate PPV and specificity, suggesting efficacy in ruling out appendicitis, it should be supplemented with radiologic findings for a definitive diagnosis. Tenderness remains a crucial clinical sign, emphasizing its importance on physical examination. These findings should guide future research to refine the MAS components and explore complementary diagnostic strategies to improve the accuracy of pediatric appendicitis diagnosis.

## Introduction

The acute abdomen, a joint emergency department presentation, poses diagnostic challenges in distinguishing between medical and surgical causes. While accurate history, focused physical examination, and fundamental investigations aid in determining the cause, radiologic support like ultrasound (US) can provide more precise diagnoses and guide treatment decisions [1]. Diagnosing acute abdominal pain in children is particularly challenging due to generalized pain and difficulty in localizing tenderness. Appendicitis, a severe surgical emergency in pediatrics, typically presents with pain originating from the umbilicus and potentially radiating to the right iliac fossa [2]. Delayed diagnosis can lead to increased morbidity and mortality due to inflammation progression and potential bowel perforation [3]. Vague symptoms in pediatric abdominal pain cases may require monitoring for disease progression and possible surgery [4].

To minimize unnecessary appendectomies, various recommendations include predictive scoring systems, clinical assessment, inflammatory markers, and radiologic support such as US and computed tomography (CT). Clinical diagnosis of appendicitis often employs scoring systems like the pediatric appendicitis score (PAS) and the widely used Alvarado score or modified Alvarado scoring system (MAS) [5]. There is ongoing controversy regarding the accuracy of these scoring systems and supporting laboratory parameters, with some studies questioning their reliability in certain patient populations or clinical scenarios [6]. Despite this, they have been introduced to aid diagnosis and reduce false-positive appendectomy rates [7]. The MAS, based on signs, symptoms, and laboratory data, helps risk-stratify patients with suspected appendicitis and may reduce the need for high-radiation computed tomography (CT) scans [8].

US is often the first-choice radiologic method for diagnosing acute appendicitis, though its effectiveness can vary based on operator skill and patient factors. The American College of Radiology recommends radiologic studies for children with moderate to high-risk scores [9]. However, it is important to note that CT is recommended when clinical difficulties persist and repeat US is inconclusive. CT scans, while effective, do carry the risk of radiation exposure, especially in pediatric patients [10].

Literature comparing appendicitis scoring systems has demonstrated the potential of the MAS in radiologic studies, combined with other methods and independently. One study found MAS to have higher sensitivity (95%) and predictive value (90) compared to US (89.5% sensitivity, 77% predictive value) [11]. While most studies focus on the efficacy of radiologic studies and the MAS alone or in combination [12], there is limited literature directly comparing radiologic examinations and MAS in diagnosing appendicitis. This study aims to compare the efficacy and diagnostic accuracy of MAS and radiologic examinations in diagnosing acute appendicitis, with the hopeful hypothesis that combining both methods may yield high sensitivity and specificity.

## Materials and methods

Study design and setting

This retrospective comparative study was conducted at Prince Mohammad Bin Abdulaziz Hospital, Madinah, Saudi Arabia, from January 2018 to December 2020. The institutional review board approved the study, and due to its retrospective nature, the requirement for formal consent was waived.

Population and sample size

The study included pediatric patients under the age of 14 who presented to the emergency department with acute abdominal pain suggestive of a surgical abdomen, particularly with suspected acute appendicitis. A total of 177 pediatric patients who met the inclusion criteria were included in the study. Given the prevalence of appendicitis in the pediatric population, the sample size was calculated to ensure sufficient statistical power.

Sampling procedure

Purposive sampling was used to select pediatric patients with symptoms suggestive of acute appendicitis. The clinical examination included a detailed medical history, physical examination, laboratory tests, and radiologic imaging (US and CT).

Inclusion and exclusion criteria

The inclusion and exclusion criteria for the study are summarized in Table 1 (Adapted from study protocol).

**Table 1:** Inclusion and exclusion criteria. MAS: modified Alvarado scoring system.

Inclusion criteria	Exclusion criteria
Pediatric patients aged less than 14 years	
Presentation with acute abdominal pain, particularly pain migrating from the umbilicus to the right iliac fossa	Abdominal pain attributable to causes other than appendicitis
Initial clinical impression of possible appendicitis based on history and physical examination	Previous abdominal surgeries complicating the diagnosis of appendicitis
	Incomplete medical records or missing data on the MAS or radiologic findings

Data collection

Data were extracted retrospectively from the hospital's medical records using a standardized data collection form. Information collected included patient demographics (age and gender), clinical symptoms (migration of pain, anorexia, nausea, vomiting), physical examination findings (tenderness, rebound tenderness, elevated temperature), and laboratory findings (leukocytosis). The MAS was calculated for each patient based on clinical data, with scores categorized as low risk (1-3), intermediate risk (4-6), or high risk (7-9). Radiologic findings were documented, including US and CT scan results.

Data analysis

The collected data were analyzed using IBM SPSS Statistics for Windows, Version 25.0 (Released 2017; IBM Corp., Armonk, New York, United States). The descriptive statistics summarized demographic data, clinical findings, MAS, and radiologic results. Categorical variables were presented as frequencies and percentages, with the corresponding sample sizes (n) in the format n (%). Continuous variables were presented as means and standard deviations.

The chi-square test was used to assess the association between MAS and radiologic findings for comparative analysis. Sensitivity, specificity, positive predictive value (PPV), negative predictive value (NPV), and accuracy were calculated for MAS, radiologic studies, and the combination of both modalities. All statistical tests were two-sided, and a p-value of less than 0.05 was considered statistically significant.

Quality assurance

To ensure the accuracy and reliability of the data, the following quality assurance measures were performed: Data collection was standardized using a uniform datasheet to minimize variability and errors. Two researchers independently reviewed the clinical and radiologic data to ensure consistency and accuracy in MAS calculation and interpretation of radiologic findings. An experienced biostatistician performed a statistical analysis to ensure the validity of the results.

## Results

A total of 177 patients were enrolled in this study, 92 (52%) men and 85 (48%) women. The common age was 7-10 years (95, 53.7%).

Comparing the MAS with positive radiology, we found that of the 177 cases, 46 (26%) children had positive radiologic findings for appendicitis, 18 (10%) children had MAS of ≥7, 24 (14%) had a scores of 4-6, and only 4 (2%) with MAS ≤3. For those who were negative for radiologic findings, i.e., 131 (74%) for appendicitis, we found that 19 (11%) had scores ≥7, 70 (39%) had scores 4-6, and 42 (24 %) had scores <3.

We also found a significant difference between the MAS in positive and negative appendicitis groups using a chi-square test with a p value of 0.001 (a p-value less than 0.05 was considered significant) (Table 2). The MAS sensitivity was found to be 81%, specificity 69%, PPV 48%, and NPV 91% (Table 2).

**Table 2:** Modified Alvarado score. RLQ: right lower quadrant. Scores: 1-3, Low risk (unlikely); 4-6, moderate risk (possible); 7-9, high risk (definite).

Signs and symptoms	Score
Migratory right iliac fossa pain	1
Anorexia	1
Nausea and vomiting	1
Tenderness of RLQ	2
Rebound tenderness of right iliac fossa	1
Elevated temperature	1
Leukocytosis	2
Modified Alvarado score	9

Comparing the radiologic studies and MAS simultaneously, we found a PPV of 0.39 and a NPP of 0.14, with a sensitivity of 0.48 (48%) and a specificity of 0.80 (80%). The accuracy was 0.73, with a positive likelihood of 2.4, a negative likelihood of 2.4, and an odds ratio of 3.69 (Table 3).

**Table 3:** Sensitivity, specificity, PPV, and NPV of MAS (individual) components. PPV: positive predictive value; NPV: negative predictive value; MAS: modified Alvarado scoring system; WBCs: white blood cells.

	Sensitivity (%)	Specificity (%)	PPV (%)	NPV (%)
MAS	81	69	48	91
Radiologic studies	41.3	100	100	82.9
Rebound tenderness	50	83	52	82
WBCs	71	54	55	84

The study also identified that, of 177 patients, 46 (26%) had positive radiologic findings for appendicitis. Of these, 23 (13%) had rebound tenderness on the physical exam and 23 (13%) had no or minimal tenderness. Among the 131 (74%) patients with negative radiologic findings for appendicitis, 21 (12%) had rebound tenderness on physical examination and 110 (62%) had no tenderness. There was a significant difference between the physical exam in positive and negative appendicitis groups using the chi-square test with a p value of 0.001 (p-value less than 0.05 was considered significant in all statistical tests) (Table 4).

**Table 4:** Correlation of MAS and radiology for appendicitis. MAS: modified Alvarado scoring system.

Alvarado score	Appendicitis (+)	Appendicitis (-)	p-value
1-4	4 (2%)	42 (24%)	0.001
5-6	24 (14%)	70 (39%)	
7-9	18 (10%)	19 (11%)	

Table 5 displays the distribution of signs and symptoms related to the MAS in our study group, categorized by the final diagnosis of appendicitis. This analysis offers insight into the diagnostic significance of the individual MAS components. Specifically, we found that wandering right lower quadrant (RLQ) pain exhibited a notable difference (p = 0.02) between patients with and without appendicitis, with 7.9% of appendicitis cases experiencing this symptom compared to 11.3% of non-appendicitis cases. The appendicitis group was more likely to have tenderness (13.0% versus 11.9%). Furthermore, an elevated white blood cell (WBC) count demonstrated a highly significant association with appendicitis (p = 0.002), occurring in 18.6% of appendicitis cases versus 33.3% of non-appendicitis cases.

Interestingly, anorexia/irritability and nausea/vomiting did not show statistically significant differences between the two groups (p = 0.1 and p = 0.5, respectively). These findings indicate that specific MAS components, mainly migratory RLQ pain and elevated leukocytes, hold significant predictive value for appendicitis in our pediatric population. On the other hand, some elements may be less reliable indicators. This highlights the importance of considering MAS components alongside other clinical and diagnostic parameters when assessing suspected appendicitis in children.

**Table 5:** Signs and symptoms of MAS in the sample. MAS: modified Alvarado scoring system; WBCs: white blood cells.

Signs and symptoms of MAS		Appendicitis (-)	Appendicitis (+)	p-value
Migratory RLQ pain	Yes	11.3%	7.9%	0.02
	No	62.7%	18.1%	
Rebound tenderness	Yes	62.1%	13.0%	
	No	11.9%	13.0%	
WBCs	Yes	33.3%	18.6%	0.002
	No	40.7%	7.3%	
Anorexia/irritability	Yes	35.8%	15.3%	0.1
	No	38.1%	10.8%	
Nausea/vomiting	Yes	65.0%	23.2%	0.5
	No	9.0%	2.8%	

## Discussion

The main findings of our study were the children who were radiologic positive for appendicitis but had an MAS of only ≤3, while 14% radiologically significant had an MAS of 4-6. Similarly, those with radiologically insignificant findings but a high MAS may be due to other causes of acute abdomen, i.e., 11% with a score >7. The accuracy of radiologic studies has variable outcomes with a sensitivity of 98% (range of 44-100%) and specificity of 83% (range of 47-99%) in diagnosing acute appendicitis. [13].

Clinical sensitivity and specificity for appendicitis are limited, may delay the diagnosis, and are directly related to an increased risk of mortality and morbidity, particularly among children [14]. Shreef et al. reported right iliac fossa pain in 52% of cases who were falsely operated for appendicitis [10]. We found that 63% of cases with low Alvarado scores (≤6) did not have acute appendicitis radiologically [15]. We also found that 26% of cases had positive radiologic findings for appendicitis, and 13% had rebound tenderness on physical examination. In comparison, 74% were negative on radiology for appendicitis, although 12% had rebound tenderness on physical examination with a significant p value (0.001).

Nasiri et al. [16] observed 10.6% of patients with a normal appendix (false negative) with 4.6% false positives. Radiologic studies alone have resulted in correct diagnosis in 87%; however, with MAS alone, it was 88%. The literature demonstrates the variability of results on using radiologic studies to detect appendicitis, with a high sensitivity of 98% and a specificity of 83%. The PPV, NPV, and AUC were 95%, 94%, and 0.907, respectively. Hosseini et al. reported PPV and NPV of 58% and 68%, respectively [17]. Similarly, Abu-Yousef et al. reported a sensitivity of 80% and specificity of 95% [18], while a meta-analysis identified a sensitivity and specificity of 81% and 87%, respectively. [19].

Using MAS alone, 6% of acute appendices have been missed (false negatives), and 7% of unnecessary operations have been performed (false positives). Interestingly, when both modalities were statistically significant for appendicitis, there were 45 true positives and no false positives. When the Alvarado score was negative or equivocal, adding radiologic studies decreased the false negative rate by 75% [20]. Al-Wageeh et al. [13] identified a sensitivity of 95% and specificity of 88% in identifying appendicitis, with PPV, NPV, and AUC values of 96%, 84%, and 0.938, respectively, for MAS. However, MAS has its limitations, as elaborated by Bai et al. [21], with a cumulative sensitivity of 87% and specificity of 47% for pediatric appendicitis.

Combining the efficacy of radiologic studies and MAS as described by Kurane et al. [22], it has a sensitivity of 88.8%, specificity of 96.5%, PPV of 94%, NNP of 93%, and accuracy of 93.6%. While we have different results, this may be due to the prospective nature of this study with limited cases.

These findings suggest that MAS may not be wholly accurate in diagnosing appendicitis alone. Therefore, we may need additional radiologic support to confirm the diagnosis and prevent false-positive appendectomies. Interestingly, radiologic methods alone may lead to false-negative results. So, combined and additional methods may be required to reach the final diagnosis of appendicitis.

This study has several limitations. Its retrospective nature may introduce potential biases in data collection and interpretation. The sample size, while adequate for initial analysis, may limit the generalizability of our findings to larger populations. Additionally, as a single-center study, it may not fully represent the diversity of clinical presentations and diagnostic approaches across different healthcare settings. Future prospective, multicenter studies with larger sample sizes could help address these limitations and further validate our findings.

## Conclusions

This retrospective comparative study of 177 pediatric patients with a balanced gender distribution validates the MAS as a valuable diagnostic tool for appendicitis, particularly effective in children aged 7-10 years. Our analysis demonstrates the robust diagnostic capabilities of the MAS, with a high sensitivity of 81% and an impressive negative predictive value of 91%. This highlights its potential to significantly impact pediatric healthcare by reliably ruling out appendicitis in low-scoring cases, inspiring a new wave of diagnostic strategies in pediatric healthcare.

The study found a statistically significant correlation between elevated MAS and positive radiologic findings (p = 0.001), confirming the method's diagnostic utility. While the radiologic examinations showed optimal specificity and a PPV (100%), their significantly lower sensitivity (41.3%) revealed a critical limitation: the risk of missing significant cases as the only diagnostic tool.

A crucial result emerged from the integration of several diagnostic approaches. Combining MAS (including physical examination findings and significantly rebound tenderness findings) with radiologic examinations increased the overall diagnostic accuracy to 73%. This result underscores the importance of your role in patient care and the need for a comprehensive diagnostic strategy versus assessment by a single method. In addition, rebound tenderness showed a significant correlation with appendicitis (p = 0.001), emphasizing the importance of a thorough physical examination in the diagnostic process.

These comprehensive results suggest that although MAS is an excellent initial screening tool, especially for ruling out appendicitis, its moderate PPV necessitates the integration of complementary diagnostic methods. Implementing a systematic multimodal approach, including MAS, a thorough physical examination, and selective radiologic imaging, provides the most reliable diagnostic strategy. This integrated approach optimizes diagnostic accuracy and is critical in reducing unnecessary surgical procedures and minimizing radiation exposure through selective imaging, ultimately improving patient outcomes in pediatric appendicitis.
